# New Appliances and Things Medical

**Published:** 1895-12-14

**Authors:** 


### NEW APPLIANCES AND THINGS MEDICAL.

[We shall be glad to receive, at our Office, 428, Strand, London, W.C., from the manufacturers, specimens of all new preparations and appliances which may be brought out from time to time.]

### ROBINSON'S COCA WINE AND LIEBIG'S BEEF WINE.

Pendleton, Manchester.

Specimens of the above have been sent to us for notice. First, then, in what way do these differ from the numerous other preparations of the same name? The coca wine is made by infusing directly in sound port the leaves of the best coca for some weeks and then expressing the leaves by hydraulic pressure and adding the expressed fluid to the filtered-off port. This process gives all the aromatic and volatile principles present in the leaves, as well as those which are dissolved in the wine. Now this process differs very considerably from that ordinarily followed, which is to boil the leaves in water and add the aqueous extract to the wine, thus losing the volatile and aromatic principles which go to help the soluble constituents in their action. Robinson's coca wine, therefore, is a real vinous extract of the whole leaf, consequently the wine is much more likely to produce favourable results when used in those cases where the whole drug is required. Our analysis fully justifies the above remarks. Coca Wine: Alcohol, 15·55 per cent. by weight; total acidity low, viz., an equivalent to 17·3 c.c. of decinormal soda; volatile acidity, only 5·5 c.c.; total solid residue, 9·59 per cent. This shows a full complement of coca leaf extract. This residue yielded 0·268 per cent. of ash. This is equivalent to at least 2 grammes of coca leaves to every 100 c.c. of port wine. Next as to the beef wine. Here the manufacturer takes the trouble to extract the tannin before adding the Liebig's meat extract and malt extract, consequently there is no loss of nitrogenous constituents by precipitation, which might cause a reduction in the food and stimulant value of the wine. Analysis of Beef Wine (port wine from which all the tannin has been eliminated): Nitrogen, 0·155 per cent.; total extract 16·81 per cent., yielding 1·21 per cent. of ash. This extract equals natural solid residue of wine (less the tannin), solids of the beef extract and the solids of malt extract. The acidity of the wine is slightly higher than that of the coca sample. The alcoholic strength is equal to 14·1—rather less than that of the coca wine. The amount of beef extract being small the natural bouquet and flavour of the wine are not masked altogether. Next as to the appearance and taste of the wines. Both are clear, free from sediment, and pleasant tasting. The coca is an especially good specimen of such a wine—clean tasting and free from the musty flavour so often met with in such preparations. We have given both wines a trial with patients, and can favourably report on them. The coca wine in a case of menopause hæmorrhage, with great nervous exhaustion, enabled the patient to take other food which before she was unable to look at. The beef wine, too, has succeeded in cases of visceral disease requiring fluid nourishment, and that of a stimulating character. We can speak favourably of both preparations.

